# Supplementary material for: Optimization of EPA-Nattokinase Nanoemulsions Processed by High-Pressure Homogenization to Enhance Stability and Thrombolytic Efficacy
Source: Foods. 2025 Oct 12;14(20):3482. doi: 10.3390/foods14203482 (PMC12563103; doi:10.3390/foods14203482)
Supplement: Supplementary file 1 [file foods-14-03482-s001.zip › foods-3907221-supplementary.pdf]

Table S1 Test design factor and level value

Factor	level		
	-1	0	1
The amount of emulsifier (%)	1.5	2.0	2.5
The blending ratio of emulsifiers	3:3	4:2	5:1
The number of high-pressure homogenization cycles (cycle)	2	3	4
High-pressure homogenization pressure (MPa)	70	80	90

Table S2 The experimental design and results for response surface analysis Box-Behnken

number	A Emulsifier dosage （%）	B Compounding ratio AKO: EPC	C Homogenization times （times）	D homogenizing pressure （MPa）	Enzyme activity retention rate （%）
1	-1	-1	0	0	78.10
2	1	-1	0	0	76.19
3	-1	1	0	0	78.57
4	1	1	0	0	79.68
5	0	0	-1	-1	66.44
6	0	0	1	-1	63.54
7	0	0	-1	1	66.67
8	0	0	1	1	57.79
9	-1	0	0	-1	62.54
10	1	0	0	-1	72.38
11	-1	0	0	1	63.81
12	1	0	0	1	63.39
13	0	-1	-1	0	82.22

14	0	1	-1	0	68.57
15	0	-1	1	0	67.62
16	0	1	1	0	70.47
17	-1	0	-1	0	71.43
18	1	0	-1	0	79.52
19	-1	0	1	0	73.33
20	1	0	1	0	64.13
21	0	-1	0	-1	68.89
22	0	1	0	-1	68.57
23	0	-1	0	1	66.98
24	0	1	0	1	59.05
25	0	0	0	0	88.57
26	0	0	0	0	92.38
27	0	0	0	0	91.42
28	0	0	0	0	89.66
29	0	0	0	0	90.36

Table S3 Significance test for regression coefficient

source of variation	quadratic sum SS	degree of freedom DF	mean square MS	F-value	P-value	
Model	2785.87	14	198.99	33.61	＜0.0001	**
A- emulsifier dosage	4.700	1	4.70	0.7939	0.3880	--
B- Emulsifier ratio	18.98	1	18.98	3.21	0.0950	--
C-Homogenization times	120.14	1	120.14	20.30	0.0005	**
D- homogenizing pressure	50.72	1	50.72	8.57	0.0110	*

AB	2.2801	1	2.28	0.3852	0.5448	--
AC	74.74	1	74.74	12.62	0.0031	**
AD	26.32	1	26.32	4.45	0.0534	--
BC	68.06	1	68.06	11.50	0.0044	**
BD	14.48	1	14.48	2.45	0.1402	--
CD	8.94	1	8.94	1.51	0.2394	--
A^2	311.84	1	311.84	52.68	＜0.0001	**
B^2	291.49	1	291.49	49.24	＜0.0001	**
C^2	763.76	1	763.76	129.02	＜0.0001	**
D^2	1943.83	1	1943.83	328.36	＜0.0001	**
Residual	82.88	14	5.92			
Lack of fit	74.05	10	7.40	3.35	0.1273	--
Net error	8.83	4	2.21			
Overall error	2868.754	28				
R^2	0.9711					
R^2_{adj}	0.9422					

Note: ** indicates an extremely significant difference ($p < 0.01$); * indicates a significant difference ($p < 0.05$); -- indicates that the difference is not significant ($p > 0.05$).

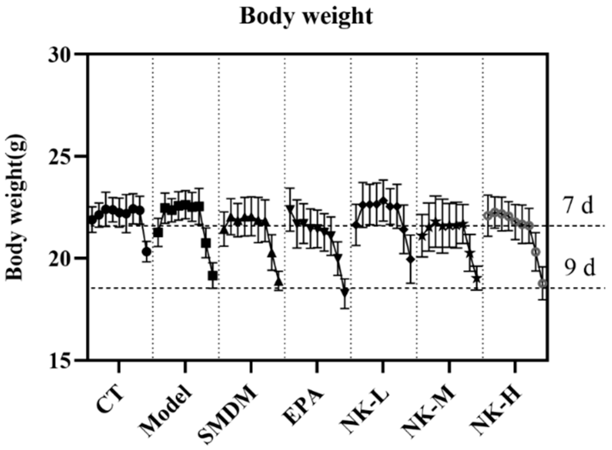

Figure. S1. Body weight change of mice.

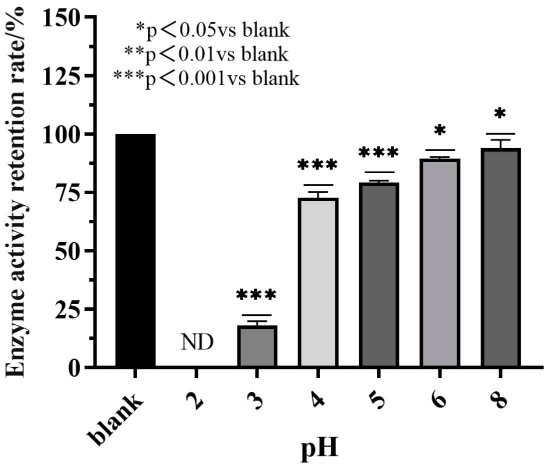

Figure. S2. The influence of pH on the fibrinolytic activity of nattokinase
